# Supplementary material for: Screening of Characteristic Metabolites in Bee Pollen from Different Floral Sources Based on High-Resolution Mass Spectrometry
Source: Foods. 2025 Dec 14;14(24):4305. doi: 10.3390/foods14244305 (PMC12732824; doi:10.3390/foods14244305)
Supplement: Supplementary file 1 [file foods-14-04305-s001.zip › foods-3971639-supplementary.pdf]

Screening of Characteristic Metabolites in Bee Pollen from Different Floral Sources Based on High-Resolution Mass Spectrometry

LIU Lanhua1, SUN Zhiwei2, LIANG Aiyuan2, ZHANG Run2, HE Siqi2, HE Yaling1, ZHANG Min2, LI Xingjiang2 * and WU Xuefeng2 *

1. Instrumental Analysis Center, Hefei University of Technology, Hefei 230009, Anhui, China

2. Anhui Fermented Food Engineering Research Center, Key Laboratory for Agricultural Products Processing of Anhui Province, School of Food and Biological Engineering, Hefei University of Technology, Hefei 230009, Anhui, China

Address correspondence to:Wu Xuefeng, Li Xingjiang 193 Tunxi Road, Hefei, Anhui Province, 230009 P.R. China

Tel: +86-551-62919371 Fax: +86-551-62901507

E-mail: wuxuefeng@hfut.edu.cn, lixingjiang@hfut.edu.cn

Supplementary Material Guide:

Figure S1. Model validation parameter graph

Figure S2. Calibration curve of polyphenol

Figure S3. Organic acid standard curve

Figure S4. Sugar standard curve

Figure S5. Ion current and mass spectrum diagrams of the characteristic metabolites of bee pollen from different flower sources.

Table S1. Data on the recovery rate of polyphenols in the solid-phase extraction (SPE) method

Table S2. Original data of volatile metabolites from different flower-source bee pollen

Table S3. Original data of non-volatile metabolites in bee pollen from different flower sources

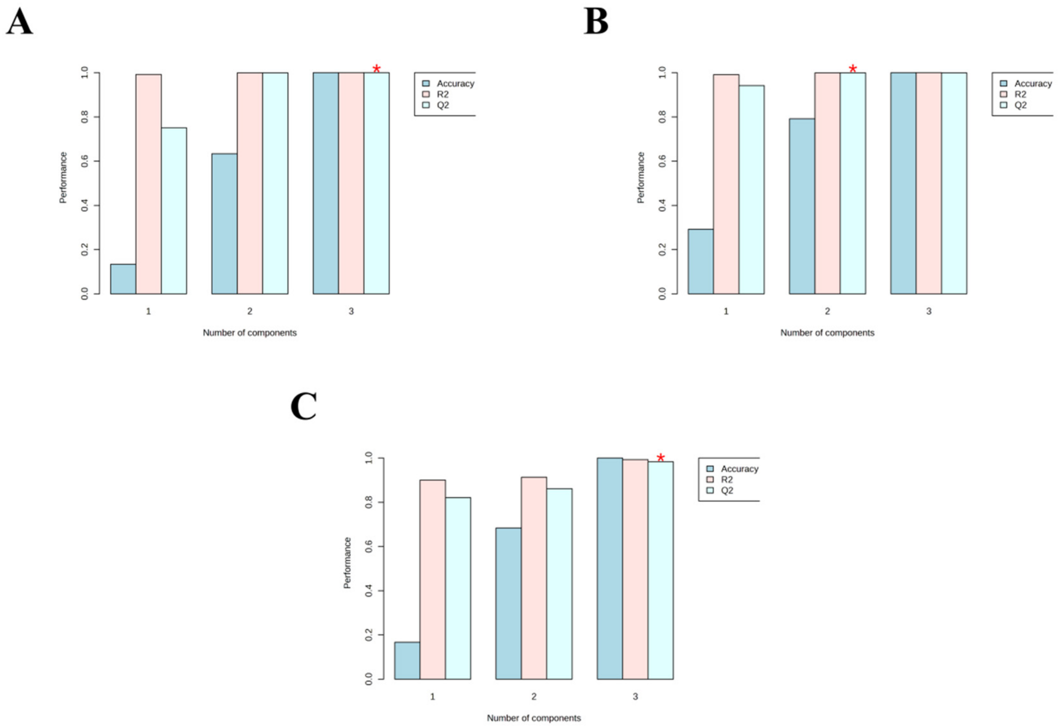

Figure S1. Model validation parameter graph. Note: Chemical components (A), Volatile metabolites (B), Non-volatile metabolites (C).

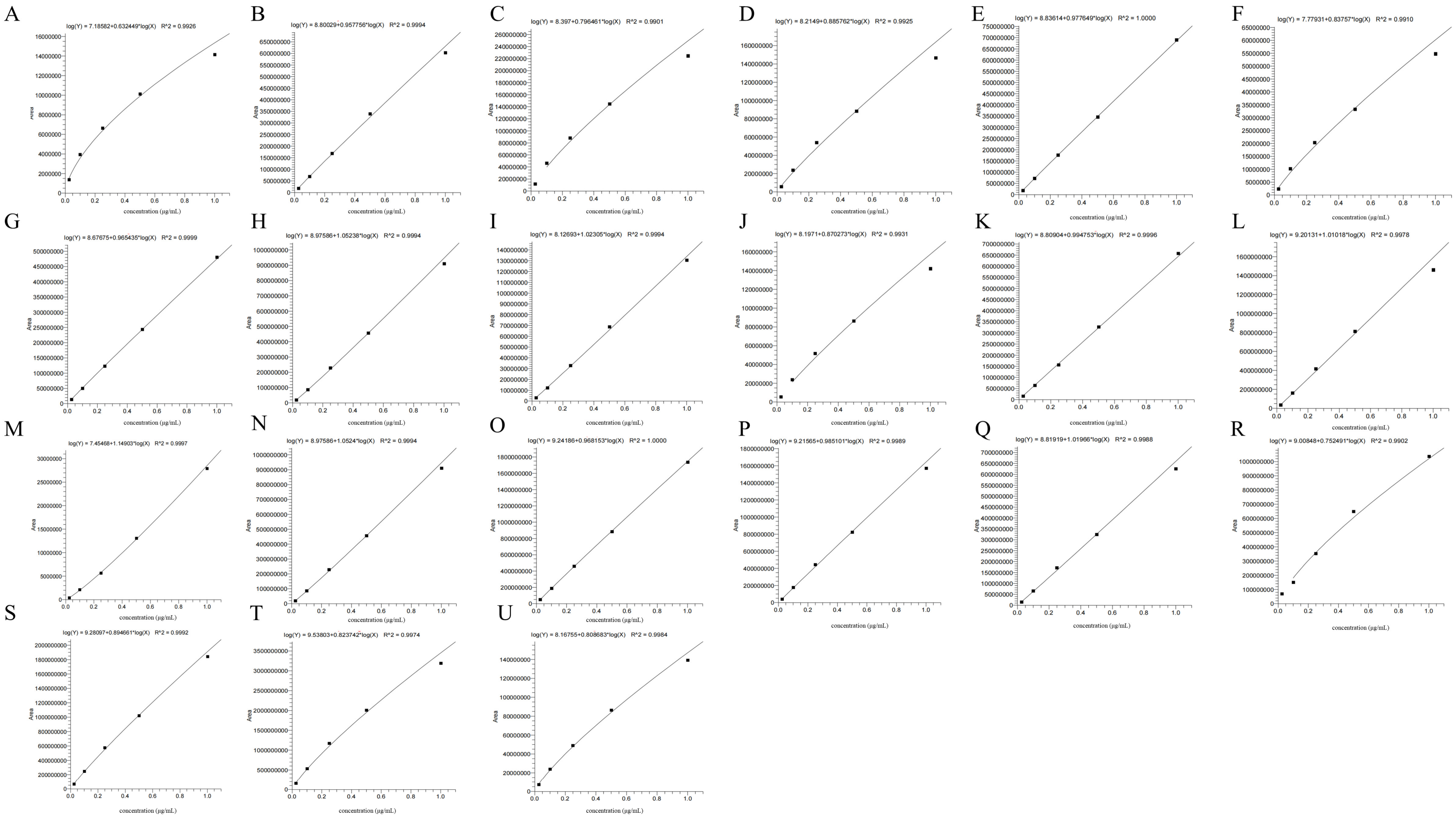

Figure S2. Calibration curve of polyphenol. Arbutin (A); protocatechuic acid (B); (+)-Catechin hydrate (C); Chlorogenic acid (D);Caffeic acid (E); (-)-Epigallocatechin gallate (F); trans-4-Hydroxycinnamic acid (G); Ellagic acid;Rutin (H); (-)-Epicatechin gallate (I); (-)-taxifolin (J); Astragalin (K); Myricetin (L);

Quercetin (M); (+/-)-Naringenin (N); Kaempferol (O); Isorhamnetin (P); Isorhamnetin (Q); Apigenin (R); Isoliquiritigenin (S);Pinocembrin (T); Ursolic Acid (U).

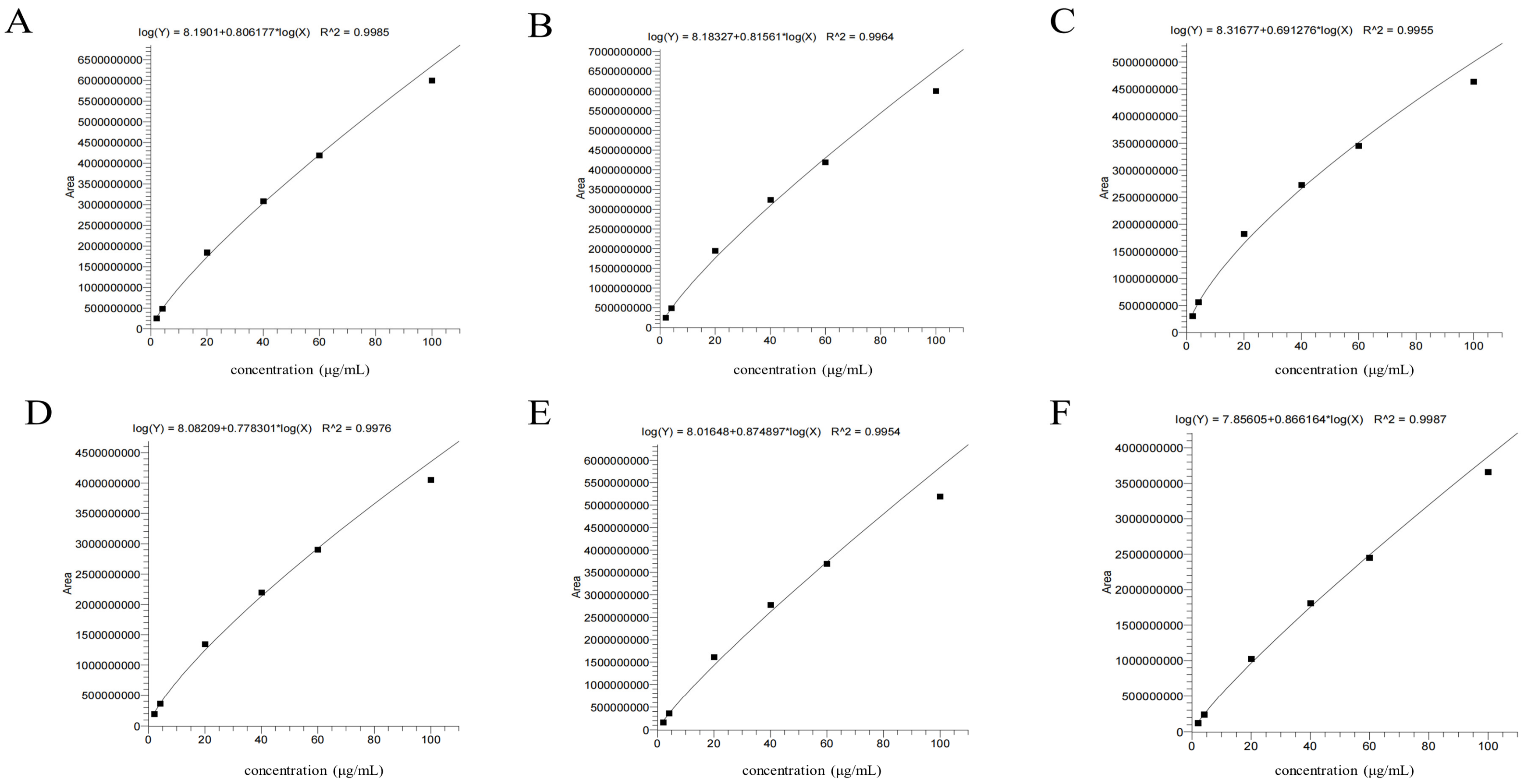

Figure S3. Organic acid standard curve. Oxalic acid (A), Tartaric acid (B), Quinic acid (C), Malic acid (D), Lactic acid (E).

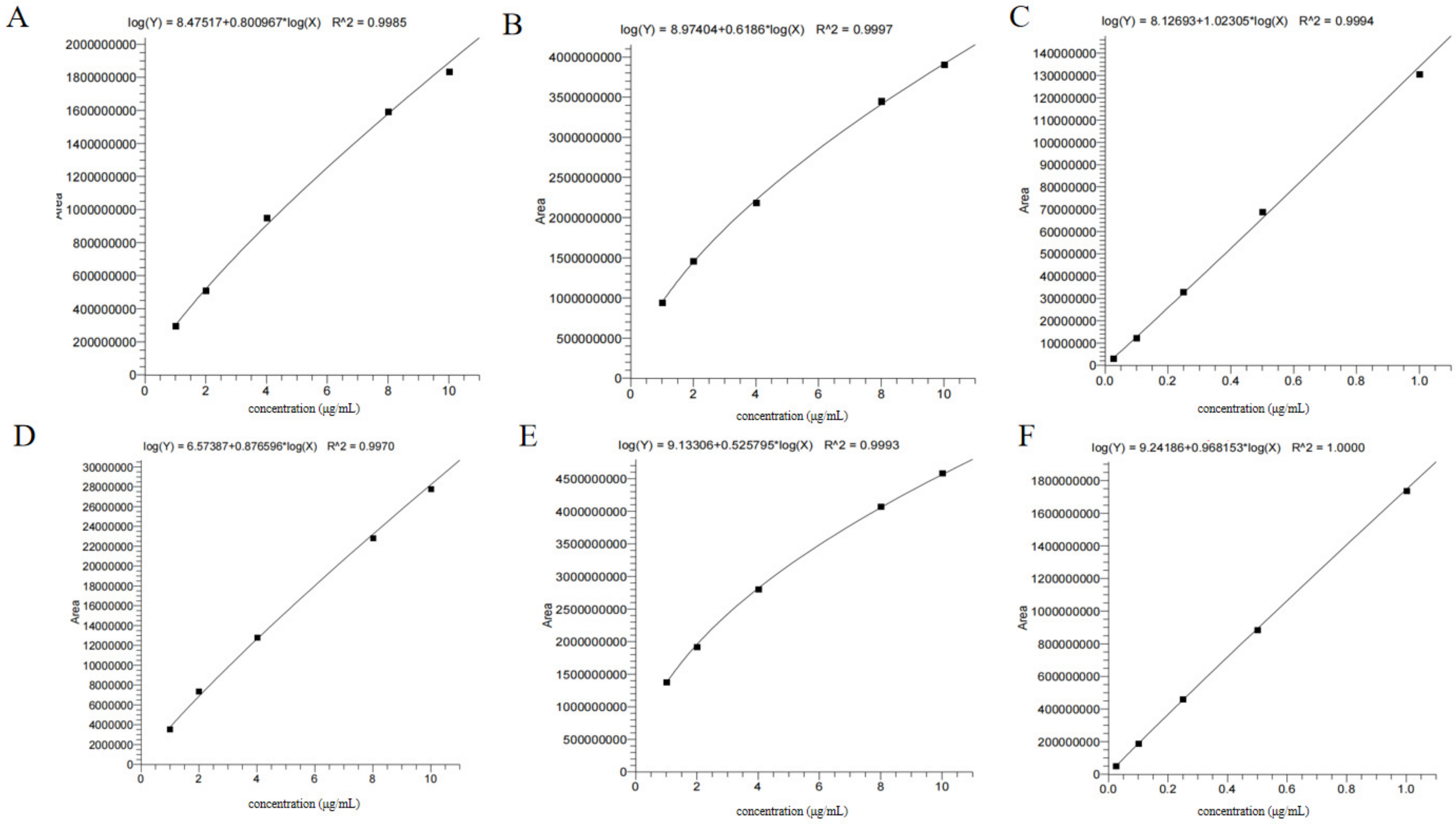

Figure S4. Sugar standard curve. Mannose (A), Sucrose (B), Fructose (C), Glucose (D), Maltose (E), Fucose (F).

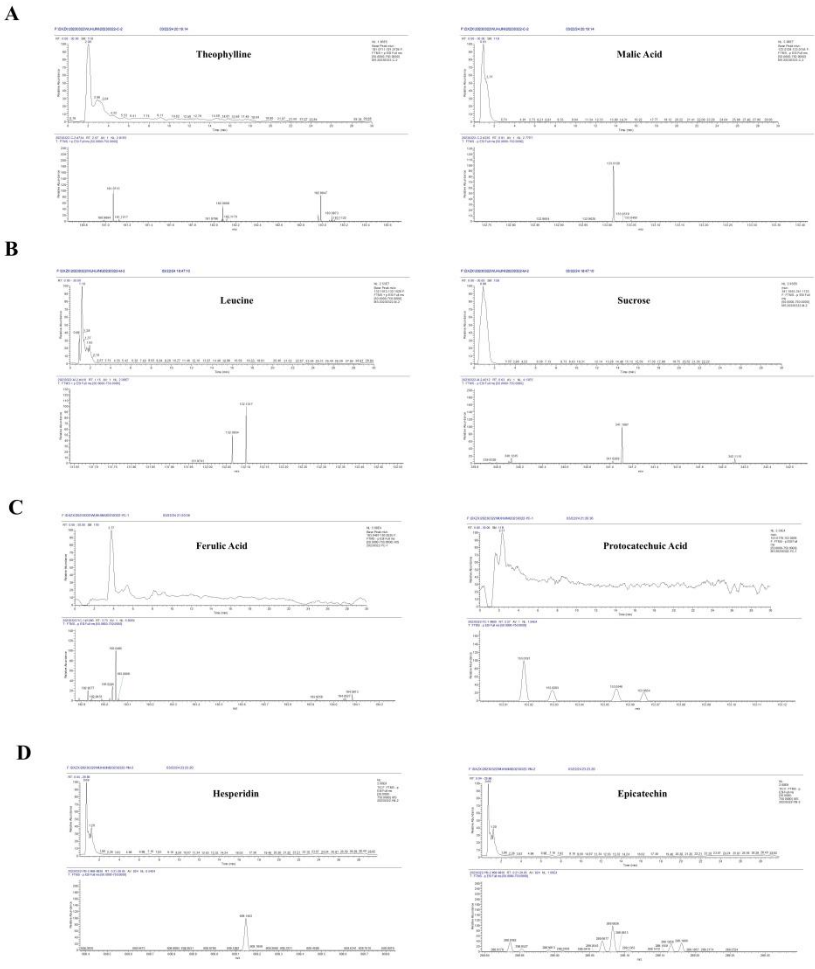

Figure S5. Ion current and mass spectrum diagrams of the characteristic metabolites of bee pollen from different flower sources. Tea pollen (A), Rose pollen (B),

Rapeseed pollen (C), Corn pollen (D)

Table S1 Data on the recovery rate of polyphenols in the solid-phase extraction (SPE) method

Compounds	recovery rate（%）
Arbutin	90.13±2.25
Protocatechuic acid	67.65±1.03
(+)-Catechin hydrate	74.43±1.38
Chlorogenic acid	81.03±1.25
Caffeic acid	64.25±0.76
(-)-Epigallocatechin gallate	71.48±0.52
Trans-4-hydroxycinnamic acid	57.12±0.58
Ellagic acid	53.48±2.57
Rutin	72.19±0.55
(-)-Epicatechin gallate	84.56±0.52
(-)-Taxifolin	79.18±0.62
Astragalin	78.88±1.68
Myricetin	73.58±0.67
Quercetin	78.53±1.48
(+/-)-Naringenin	69.82±2.44
Kaempferol	73.18±1.66
Isorhamnetin	90.47±3.62
Apigenin	71.58±1.62
Isoliquiritigenin	75.43±2.84
Pinocembrin	86.07±1.58
Ursolic acid	59.48±0.62

Table S2 Original data of volatile metabolites from different flower-source bee pollen

Compounds	Tea pollen	Rose pollen	Rapeseed pollen	Corn pollen
Eugenol methyl ether	0	5358.814204	0	0
2,6-Dimethylheptane	182922.9096	0	0	0
2-Furan carboxylic acid 4-biphenyl ester	25150.00332	0	0	0
3,8-Dimethyldecane	0	0	155371.598	213335.6654
Benzyl benzoate	3202822.797	1356729.358	0	0
Dimethoxybenzofuranone	0	107628.1095	0	0
Nerol	0	5531.123443	0	0
Butyldiolone	0	767862.9312	0	0
Eugenol	1242716.393	1806084.42	2668783.927	1942423.093
Eugenol acid	0	0	0	67736.99778
Eugenol ester	0	88103.58071	0	0
Scopolamine	0	107996.1895	0	0
Tert-butylphenol	3032840.807	770960.0282	480314.9544	398632.7606
Octylbenzoinone	50159.70165	0	0	0
Biphenyl	4277.378143	0	4450.053506	132138.5944
Twenty-nine alkanes	0	101566.1434	0	0
Twenty-seven alkanes	0	295091.3088	358854.1195	508969.8498
Twenty alkanes	0	136039.8359	0	0
Twenty-one alkanes	479320.968	0	0	0
Aromatic acid esters	0	3160.166314	0	0
Ethyl tetrahydrofuran fumarate	177248.7726	0	0	0
Decane	80033.68729	0	0	0
Hesperetin	19638.62788	0	0	0
Cycloalkanoic acid	0	139549.9784	0	0

Fenchol ether	0	20596.45655	23674.82291	29057.67504
Propyl hexanoate	0	331255.7144	0	0
Methyl hexanoate	177098.2011	0	0	0
Hexenol	0	182009.1027	0	0
Phthalate esters	24914.94308	0	0	0
Maleic anhydride	0	572442.0761	0	0
Geranium oil	0	0	17828.73313	3644.439829
Hydroxy coumarin	0	0	3852.327346	592766.4069
Dysoxylum lignin	0	0	4535.68342	75118.29611
Trimethoxycinnamyl acid ester	0	47213.93734	0	0
Octadecane	0	277324.1279	196831.3711	898961.8367
Dodecanal	153226.5347	0	0	0
Heptadecane	0	252569.6967	0	0
Alkylphenols	0	0	6263.50968	4846.502172
Ethyl butyrate	0	0	5096.773426	9435.377204
Linalool	0	164142.028	0	0
Furan ester	0	174220.7523	0	0
Isobutyl isoprene ester	0	0	140949.3911	86455.117
Isoeugenol acid methyl ester	0	23131.93725	0	0
Isocoumarin	0	5848.907542	0	0
Methyl stearate	0	0	378009.2356	2624773.186
Quercetin	5406.814845	0	0	0
Nerolidol	90225.56079	31867.07229	30635.07172	101140.3456
Plant sterol esters	18265.38373	0	0	0
Purple perillin	10354.74054	23058.33665	0	0
Palmitic acid methyl ester	0	1224976.888	747686.0239	666372.7747

Dehydroxy acid methyl ester	0	321952.7308	0	0
Sweet orange flavonoid	0	96996.95213	0	0
2-Furan methanol	0	0	98616.68731	245945.8004

Table S3 Original data of non-volatile metabolites in bee pollen from different flower sources

Compounds	Tea pollen	Rose pollen	Rapeseed pollen	Corn pollen
Epicatechin	72882619.8	0	0	0
12-hydroxy stearic acid	0	0	0	0
13-hydroxy octadecadienoic acid	70113485.67	0	0	0
13-peroxy linoleic acid	0	0	0	0
13-hydroxy octadecatrienoic acid	104530485.7	0	0	0
16-hydroxy palmitic acid	55577615.36	41282478.53	0	0
1-aminocyclohexanoic acid	8845951.202	30930811.99	0	0
1-aminocyclopentanecarboxylic acid	56732737.05	0	23005495.88	27329892.71
2,3-diacetamido-2,3-deoxy-D-glucuronic acid	0	293657255.7	0	0
Glyceric acid	30022662.71	78503538.11	59709278.62	0
2,6-dichlorotoluene	60382861.36	78890652.84	78483184.96	66026894.92
2,6-difluorobenzoic acid	0	0	0	0
2,6-difluoropyridine	0	0	0	14445960.83
2-acetamido-2,6-deoxy-D-galactose	0	0	0	0
2-butyne aldehyde	0	0	0	0
2-furanic acid	119913950.9	0	168875252.9	333921606
2-piperidine ethylamine	0	0	0	0
2-pyrrolidone	0	0	0	14314062.7
3,5-dihydroxyphenylglycine	0	0	23925679.74	0

3-aminobicyclic acid	0	0	8176709.258	12050824.41
3-aminopyrrolidine	0	0	0	0
3-hydroxy-3-methylvaleric acid	0	0	0	268714853.4
3-O-methyldopamine	0	0	0	0
4-hydroxy-D-proline	97943378.87	194735842.7	0	0
5-aminomethylimidazolonic acid	10682536.1	50449154.34	0	335185949.4
5-aminopentanoic acid	0	5538146.601	11765080.36	15591311.92
5-fluoro-2-hydroxybenzoic acid	0	55703123.19	0	20104877.35
5-hydroxymethylfurfural	215026572.7	0	280886618.3	146168989.1
5-methylisoxazole	60845610.16	0	23216266.62	0
5-pentylisophthalic acid	20181649.68	27393419.36	0	0
5-vinyl deoxyuridine	11140808.67	0	0	0
7-methylxanthine	132632684.2	0	215021769.8	0
8-hydroxy deoxyguanosine	0	4426062.128	54081025.48	12957409.69
9-peroxy octadecadienoic acid	78662295.26	46856052.88	0	0
Acetylbenzene	38989766.92	0	32134971.59	0
Acetic anhydride	254126243.7	0	20872082.85	196175470.8
Acetoacetic acid	0	0	0	0
Pyrrolidinone cyanohydrin Acrylic acid	0	109738492.7	0	0
Adenine	386836375.9	374816753.5	502864557.4	278218292.5
Adenosine	691834063.9	669963842.5	697342474.9	196302395.9
Quercetin-4'-O-rhamnoside	0	0	21699605.35	317510161.4
Epicatechin	0	14964942.31	0	0
Alanine	47779384.85	45596528.44	56888366	48965440.39
Alanyl-L-glutamine	0	8531247.51	0	0
Alanyl-L-proline	0	0	25582874	0

Allopurinol	14144980.93	8194829.017	0	3994193.931
Apigenin	0	0	0	0
Arachidonic acid	0	0	0	0
Arginine	345693253.8	261217107.7	285456282.7	0
Ascorbic acid	0	0	39741759.26	0
Asparagine	62552385.44	442077672.1	76133266.25	20215905.01
Aspartic acid	86398322.93	40207711.78	21727426.8	14604867.28
Nonyl acid Betaine	0	45569666.67	0	0
Caffeine	408225597.4	316737550	223499590.5	2549087909
Silymarin sugar	85561503.45	0	0	0
Choline	0	0	0	0
Lecithinylcholine	7898157431	9428328058	8943566406	5364106208
Lactic acid	50577826.03	454625125.9	0	1349283542
Coumarin	514181714.7	1626753855	0	1865086949
Rubber acid	0	0	0	0
Cyclopentenone	129921700.3	150601202	259469975.1	0
Cyclopentamine	115610841.6	0	0	0
Cytosine	166541335.4	222581427.7	387692847.5	0
Diacetyl	10921473.21	50772070.22	32101931.08	12888407.9
Diaminohexanedioic acid	0	0	0	0
Eucalyptol base	37991524.29	12687735.3	0	0
Xenomorphol phenol	0	0	0	0
Fructose	0	0	0	0
Fructose-arginine	0	0	1418452201	0
Fructose-lysine	0	11784780.37	0	24476683.52
Fumaric acid	0	26749251	37624422.95	23340989.21

Gallic acid	172872738.6	0	0	0
Genistein	141368325.6	0	0	0
Garlic aldehyde	0	0	0	0
Glutamic acid	108662447.8	0	0	0
Glutamine	9155366628	0	8430453447	5019010095
Glutathione	0	0	0	0
Reduced glutathione	0	0	0	0
Hydroxyacetic acid	142008005.4	0	48959809.44	0
Glycylglutamine	0	0	24875487.47	0
Glycylproline	156787523.9	32325017.13	64037313.67	26456872.43
Guanine	156233274.9	39439266.58	0	0
Guanosine	32725690.18	22905494.06	0	0
Methionine	39060403.57	73550730.69	32551780.55	52824235.1
Guanidylleucine	0	0	0	0
Histamine	0	13713809.64	0	0
Histidine	28429065.36	72639166.43	12354860.53	0
Homocysteine	0	0	0	0
L-lysine	0	0	0	10534933.98
L-leucine	0	9760755.528	0	0
Luteolin	0	0	0	0
Glucose-6-phosphate	358076432.2	112892509.2	61456055.54	15946336.37
Glucose-1-phosphate	0	0	45183372.45	0
Glutamate	4575140.571	0	0	0
Glutamine	0	0	46723662.21	0
Glutathione	0	0	29798416.45	0
Oxidized glutathione	0	0	303915277.7	0

Hydroxyacetic acid	226069426.7	326412802.2	422066663	48978165.7
Glycylglutamine	0	0	0	0
Glycylproline	31637941.82	0	0	0
Guanine	0	0	16170339.63	0
Guanosine	1140534753	1273529393	1093777525	187740537
Methionine	0	39248797.38	0	0
Guanidylleucine	0	101169228.1	0	0
Apple acid	0	0	65261525.7	0
Malonic acid	0	27336710.51	23925212.47	136587946.7
Luteolin-7-O-glucoside	30733552.68	0	0	0
Lactic acid	0	0	0	0
Lactose	76523651.69	87612093.31	229142433.7	12755018.27
Leucine	0	0	7936613.075	0
Leucylproline	399994460.4	0	309319134	142767466.2
Sapogenin	0	0	0	0
Linolenic acid	30603766.47	0	0	0
Luteolin	19697938.68	343247239.8	128785203.2	116646795.9
Lysine	109122746.1	119839539.4	139895566	0
Guanidylproline	0	0	0	0
Malic acid	0	0	6087373.703	0
Malonic acid	0	0	0	43457209.42
Rhamnoglucozyl-3-anisohexoside	0	0	0	0
N,N-dimethyl-D-glucosamide	0	0	0	31319689.18
N-acetyl-D-amino-glucose	0	0	0	6552216.582
N-acetylhistamine	0	0	0	6934754.272
N-acetyl-L-glutamic acid	0	0	6017980.533	0

N-acetylornithine	91661026.91	0	0	0
N-acetyl-β-L-hydroxyglucosamine	0	0	0	0
Nicotinic acid	69751166.5	91629765.75	0	41573083.56
N-methyl-leucine	10636391.16	0	4995969.475	0
N-methylglutamic acid	0	19006728.21	0	14477253.57
Deomelin	0	90189072	383657397.4	0
Oleic acid	25581699.15	0	0	0
Pantothenic acid	0	0	0	0
Phenylalanine	0	0	0	0
Proline	5127995346	3687133637	2670518942	3652756907
Prolyl-leucine	20984100.95	5392706.717	10157463.28	13618558.11
Prolyl-proline	14346764.43	6272665.715	12928335.41	10296834.18
Caffeic acid	33117652.54	0	0	0
Caffeoylglutamic acid	210568518.2	244403294.4	52192318.76	52111349.33
Pyruvic acid	0	0	0	0
Quercetin	0	0	0	33560906.37
Quercetin-3-rhamnosyl-galactoside	0	0	0	0
Quercetin-3-anisohexoside	0	53753651.74	1198974084	0
Quercetin-3-new orange sugar	0	0	0	152195183.8
Quercetin-3-β-D-glucoside	0	0	0	35717897.13
Quinic acid	13130516.22	16650805.63	0	0
Rhamnosyl-3-galactoside	0	0	0	37879643.22
Aniside	0	0	0	32796151.21
Safinol	0	0	18206380.5	0
Serine	0	0	0	0
Spermidine	47551574.24	41839444.81	50811601.21	46695683.34

Stearoylamine	19627276.5	0	0	8001166.865
Stearic acid	0	0	0	0
Succinic acid	0	0	0	0
Quercetin	494219042.8	0	447890876	242200071
Quercetin-3-rhamnosyl-galactoside	618148833	0	112700133.1	0
Quercetin-3-anisohexoside	63161241.43	2834366317	47515480.24	38620878.84
Quercetin-3-new orange sugar	30537684.93	21959617	36295083.19	24566255.53
Quercetin-3-β-D-glucoside	66178821.48	0	0	0
Quinic acid	0	0	48063915.44	69195340.45
Rhamnosyl-3-galactoside	0	0	0	49616171.13
Aniside	0	0	19109353.39	0
Sugars	0	0	2491534158	196967903.8
Safinol	384992847.8	132912035.9	307989246.8	0
Serine	84418832.49	46851169.23	72239503.29	0
Spermidine	0	0	6372739.618	0
Stearoylamine	0	0	0	0
Stearic acid	0	0	7060997.656	7259556.909
Succinic acid	490087008.8	282229106.1	360925540.6	73529146.66
Theophylline	0	0	0	0
Sucrose acid	0	12081469.87	0	0
